# The effect of epidural analgesia on cancer progression in patients with stage IV colorectal cancer after primary tumor resection: A retrospective cohort study

**DOI:** 10.1371/journal.pone.0200893

**Published:** 2018-07-20

**Authors:** Ying-Hsuan Tai, Wen-Kuei Chang, Hsiang-Ling Wu, Min-Ya Chan, Hsiu-Hsi Chen, Kuang-Yi Chang

**Affiliations:** 1 Department of Anesthesiology, Taipei Veterans General Hospital, Taipei, Taiwan; 2 Department of Anesthesiology, Shuang Ho Hospital, Taipei Medical University, New Taipei City, Taiwan; 3 School of Medicine, National Yang-Ming University, Taipei, Taiwan; 4 Department of Anesthesiology, School of Medicine, College of Medicine, Taipei Medical University, Taipei, Taiwan; 5 Department of Surgery, Taipei Veterans General Hospital, Yuli Branch, Hualien, Taiwan; 6 Department of Technology Application and Human Resource Development, National Taiwan Normal University, Taipei, Taiwan; 7 Division of Biostatistics, Graduate Institute of Epidemiology and Preventive Medicine, College of Public Health, National Taiwan University, Taipei, Taiwan; University of South Alabama Mitchell Cancer Institute, UNITED STATES

## Abstract

Retrospective clinical studies showed perioperative epidural analgesia (EA) was associated with better postoperative oncologic outcomes in patients with specific types of non-metastatic cancers. This study aimed to investigate the effects of EA on cancer prognosis after surgical intervention for stage IV colorectal cancer. In this retrospective study, patients with stage IV colorectal cancer undergoing primary tumor resection and metastasectomy between January 2005 and December 2014 were classified into two groups based on their use of perioperative EA or not and evaluated through August 2016. Primary and secondary endpoints were postoperative progression-free survival (PFS) and overall survival (OS), respectively. A total of 999 patients were included and 165 (16.5%) of them received EA. The median follow-up interval was 17.5 months and no significant difference in PFS or OS was noted between the EA and non-EA groups in the univariate analysis. Multivariable Cox proportional hazards model identified four independent risk factors both for disease progression and mortality, including American Society of Anesthesiologists (ASA) physical status ≥ 3, higher pretreatment carcinoembryonic antigen (CEA), multiple distant metastases, and pathologic lymphovascular invasion. After adjustment for the selected risk factors, the effects of EA on PFS and OS remained non-significant (hazard ratio: 1.06, 95% CI: 0.87 to 1.29, for PFS and 0.90, 95% CI: 0.68 to 1.20 for OS). Similar findings were demonstrated by propensity score analysis. Our results did not support the association between perioperative epidural analgesia and better progression-free or overall survival in patients following stage IV colorectal cancer surgery.

## Introduction

Although elective bowel resections in non-obstructed patients with stage IV colorectal cancer (CRC) is a source of continuing debate, resection of the primary tumor had been reported to be a positive prognostic factor for survival in patients with stage IV disease [1]. Selected patients with stage IV CRC are amenable to potentially curative metastasectomy, and five-year survival rates of approximately 40 percent are reported, particularly for isolated liver metastases [2]. Profound improvements in the outcomes of patients with metastatic CRC over the past 15 years have been attributed to increased use of hepatic resection in selected patients and more effective chemotherapy [2].

Although surgical excision of a primary tumor presents an opportunity to eradicate cancer or arrest its progression, it is also believed to initiate micrometastases via circulating tumor cells or activate dormant pre-existing micrometastases [3]. Perioperative immune competence is an important determinant in eradicating the residual disease and major surgery may induce a neuroendocrine and cytokine stress response, which induces transient suppression of cell-mediated immunity [4]. Besides, volatile anesthetics have been found to induce apoptosis in human T-lymphocytes in a dose-dependent pattern [5] and protect human colon cancer cells against apoptosis in vitro [6]. Prior studies also suggested opioids may suppress cell-mediated immunity, including natural killer cell cytotoxicity [7] and promote tumor growth by activating the mu-opioid receptor (MOR) [8]. In contrast, regional anesthesia may attenuate the neuroendocrine stress response and reduce opioid and intraoperative volatile anesthetic requirements to preserve host immunity and possibly lower the incidence of cancer recurrence or progression [9].

Retrospective studies suggested that epidural anesthesia and analgesia (EA) might be associated with improved overall but not recurrence-free survival in patients after surgical resection of non-metastatic cancer [10]. However, the effect of EA on the oncologic outcomes in patients receiving surgery for metastatic disease is relatively unexplored. We conducted this retrospective cohort study to evaluate the associations between perioperative EA and cancer progression or all-cause mortality in patients with stage IV colorectal cancer after surgical intervention. The effects of other major prognostic factors were also assessed simultaneously to identify the significant predictors of surgical outcomes after metastatic colorectal cancer surgery.

## Materials and methods

### Setting and patient selection

This study was approved by the Institutional Review Board (IRB-TPEVGH No. 2015-11-010CC) of Taipei Veterans General Hospital, Taipei, Taiwan. The Institutional Review Board waived written informed consent, and all the study materials were anonymized and de-identified before analysis.

**Fig 1:**
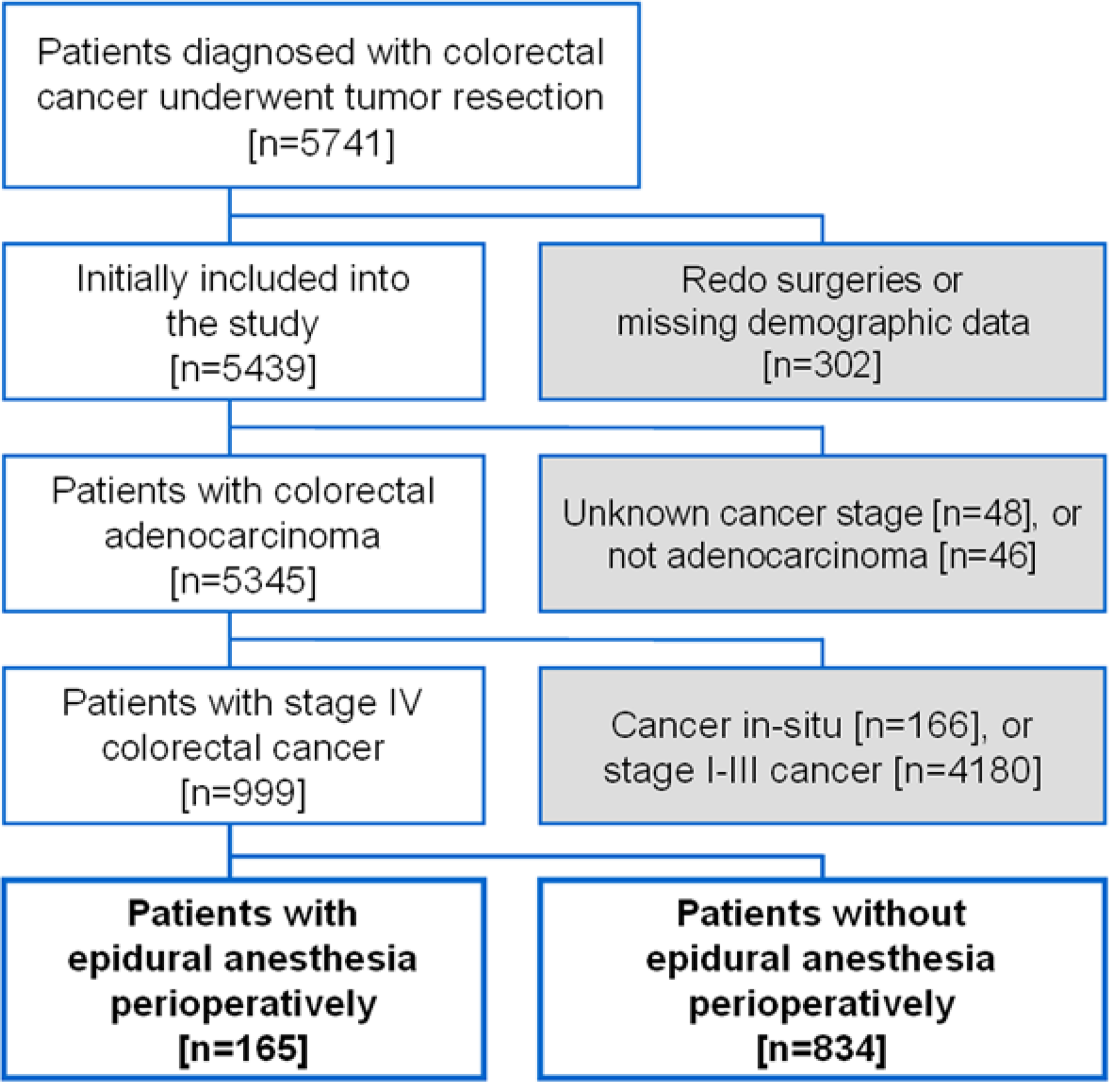
Flow diagram for patient inclusion.

We used our electronic medical database to identify patients who had primary tumor and metastatic lesion resection for stage IV colorectal adenocarcinoma from January 1, 2005, to December 31, 2014. Reoperations for disease progression were excluded from the analysis. Patients with missing data about demographics, pathologic details or postoperative analgesic were also excluded. (Fig 1) All included cases were further classified into two groups: those receiving perioperative EA and the other group without EA.

### Anesthetic management

During the study period, general anesthesia typically included fentanyl 1–2 μg·kg^-1^ and propofol 1–2 mg·kg^-1^ for induction, and neuromuscular antagonism to facilitate tracheal intubation with rocuronium 0.8 mg·kg^-1^ or cisatracurium 0.2 mg·kg^-1^. Anesthesia was maintained with sevoflurane 2–3 vol% or desflurane 6–8 vol% in oxygen, with a fraction of inspired oxygen of 0.3–0.5 at the anesthesiologist’s discretion. If epidural analgesia was selected, epidural catheters were typically inserted preoperatively at a low thoracic level (e.g., T10–T12) and assessed its function with a test dose of local anesthetic preoperatively. If an epidural was ineffective perioperatively, an intravenous patient-controlled analgesia was administered via an ambulatory infusion pump (Gemstar^™^ Yellow, Hospira, IL, USA) programmed to deliver morphine at a demand dose of 1 mg with a lockout time of 6 minutes. Epidurals were administered with a preemptive dose of local anesthetic (lidocaine 1% or 2%) with or without fentanyl 50 μg given before the surgical incision, followed by a continuous infusion of local anesthetic (bupivacaine 0.25% or 0.5%) and fentanyl 5 μg·ml^-1^ at a rate of 5–10 ml·hour^-1^ based on patients’ hemodynamics. Epidural infusion of diluted local anesthetic solution is then continued for 48–72 hours after surgery. Patients did not receive EA for a variety of reasons, including the presence of contraindications to EA and preference of anesthetists, surgeons, or patients. Patients without EA mostly received postoperative intravenous patient-controlled analgesia.

### Postoperative cancer control

Colorectal cancer staging was performed according to the American Joint Committee on Cancer 2010 TNM cancer classification system [11]. Tumor location was divided into right-sided colon (cecal to splenic flexure), left-sided colon (splenic flexure to sigmoid) or rectum. At our hospital, additional surgeries or procedures after primary tumor resection were selected on the basis of disease extent and location, including pulmonary or hepatic metastasectomy, transarterial embolization or radiofrequency ablation for liver metastases, etc. Patients included in this study received preoperative adjuvant radiotherapy or chemotherapy (Folfox- or irinotecan-based) with or without target therapy (antivascular endothelial growth factor or epidermal growth factor receptor-based) at the discretion of surgeons and patients, and was defined as any therapy given within 90 days of surgery. Standard surveillance was regularly performed after resection surgery for metastatic colorectal cancer, including serum carcinoembryonic antigen (CEA) measurement every 3 to 6 months for at least 2 years. For colon cancer, abdomen and chest computed tomography (CT) scans was performed every 3 to 6 months for 2 years, then every 6 to 12 months for 3 to 5 years. For rectal cancer, pelvis CT was added every 3 to 6 months for 2 years, then every 6 to 12 months for 3 to 5 years.

### Data collection

To determine the baseline variables and risk factors for cancer progression and mortality, we used the electronic medical database to collect demographic characteristics, pre-treatment CEA level [12], amount of packed red blood cell (pRBC) transfusion [13], pathologic features (tumor differentiation [14], mucinous or signet-ring histology [15], lymphovascular invasion [16], and perineural invasion [17]) and whether preoperative or postoperative adjuvant chemotherapy or radiotherapy was used. Current status for each patient was determined by documentation of follow-up visits to the hospital’s outpatient clinic or subsequent admissions. Relevant comorbidities were also obtained from medical records, including diabetes mellitus, coronary artery disease, heart failure, cerebrovascular disease (stroke or transient ischemic attacks), and chronic kidney disease. The radiologists and colorectal surgeons of our hospital determined whether cancer progressed or not, which was mainly based on imaging studies (CT, magnetic resonance imaging, bone scan, etc) and defined by response evaluation criteria in solid tumors (RECIST) guidelines [18]. The date of death was determined based on medical records or death certificate.

Data was extracted by specialist anesthesiologists who were not involved in data analysis. The quality of the extracted data was verified through random sampling by the authors. Data were collected up to the end of August 2016. The primary endpoint was progression-free survival (PFS), which was defined as time from the date of surgery to the date of cancer progression. The secondary endpoint was overall survival (OS), defined as time from the date of surgery to the date of death. For those without the event of cancer progression or death, their survival times are regarded as the corresponding censored observations.

### Data analysis and statistics

Demographic characteristics and pathologic findings were compared between the EA and non-EA groups using chi-square tests, Student’s t tests or Wilcoxon rank sum tests as appropriate. The cumulative incidences of cancer progression and mortality were illustrated with Kaplan-Meier method and compared between groups using log rank test. The effects of collected variables on the risk of progression or mortality were presented as hazard ratio (HR) and calculated using univariate Cox regression model. Significant predictors of progression or mortality in the univariate analysis were employed as candidates for the following forward model selection processes in the multivariable analysis. The significance level of entry criterion was set at 0.05 to identify independent risk factors of cancer progression or mortality in the multivariable analysis. The effects of EA on cancer progression and mortality were also evaluated with the adjustment for the independent predictors selected in the multivariable analysis.

Since the potential imbalance in measured variables may confound the effect of EA on cancer progression or survival, propensity scores developed from a collection of patient characteristics (age, sex, pretreatment CEA level, cancer stage, pathologic findings and so on) was used to estimate the probability of receiving EA (Appendix) and adjusted for as a covariate in the Cox regression analysis [19]. Moreover, all patients were classified into five equal-size groups based on the quintiles of the estimated propensity score and stratified Cox regression analysis was performed to calculate a pooled hazard ratio across the five strata for stage IV CRC progression or survival. A *p* value less than 0.05 was considered statistically significant. SPSS Statistics 18.0 (SPSS Inc., Chicago, IL, USA) was used for all analyses. According to Schoenfeld’s formula for the sample size estimation of proportional-hazards model [20], at least 628 subjects were needed to attain a power of 0.8 assuming a type I error rate of 0.05, relative hazard of 0.740 [21] and the proportion of patients receiving EA in this study (16.5%), and we collected more than 1.5 folds the minimum requirement to increase the statistical power of our study.

## Results

A total of 999 patients were included in this study and 165 (16.5%) of them received EA. The median follow-up period was 18.3 months (interquartile range 6.8–35.5 months) in the EA group and 17.4 months (7.6–31.0 months) in the non-EA group. No significant difference in the distributions of baseline characteristics between the two groups was found except that patients in the EA group were less likely to have neoadjuvant chemotherapy and/or radiotherapy (*p* = 0.01, Table 1) and target therapy (*p* = 0.002). Table 2 shows the details of cancer stages and pathologic features of the two groups. Note that patients in the EA group were less likely to have pathologic perineural invasion (*p* = 0.02).

**Table 1:** Patient demographics.

	EA (n = 165)	Non-EA (n = 834)	*p*
**Age, year**	65 ± 13	65 ± 14	0.889
**Sex, male**	112 (67.9%)	500 (60.0%)	0.056
**BMI, kg·m**^**-2**^	23.7 ± 3.9	23.0 ± 3.5	0.080
**ASA ≥ 3**	60 (36.4%)	327 (39.2%)	0.493
**Comorbidites**			
Diabetes	33 (20.0%)	170 (20.4%)	0.911
Coronary artery disease	11 (6.7%)	61 (7.3%)	0.769
Heart failure	10 (6.1%)	34 (4.1%)	0.256
Stroke	9 (5.5%)	48 (5.8%)	0.879
Chronic kidney disease	15 (9.1%)	123 (14.7%)	0.054
**Pretreatment CEA, μg·L**^**-1**^	19.7 (3.5–82.8)	18.4 (4.1–92.0)	0.632
**Tumor location**			0.270
Right-sided colon	48 (29.1%)	265 (31.8%)	
Left-sided colon	61 (37.0%)	338 (40.5%)	
Rectum	56 (33.9%)	231 (27.7%)	
**Anesthesia time, min**	300 (255–390)	315 (255–390)	0.567
**pRBC transfusion**			0.350
No transfusion	101 (61.2%)	476 (57.1%)	
≦4 units	46 (27.9%)	259 (31.1%)	
> 4 units	18 (10.9%)	99 (11.9%)	
**Preoperative C/T ± R/T**	15 (9.1%)	140 (16.8%)	0.013
**Postoperative C/T (< 90 days)**			0.002
Nil	13 (7.9%)	97 (11.6%)	
Pure C/T	89 (53.9%)	328 (39.3%)	
C/T + TT	63 (38.2%)	409 (49.0%)	
**Postoperative R/T (< 90 days)**	24 (14.5%)	86 (10.3%)	0.112
**Follow-up time, months**	18.3 (6.8–35.5)	17.4 (7.6–31.0)	0.370

Values were mean ± SD, counts (percent), or median (interquartile range). Continuous variables are analyzed with Wilcoxon rank-sum tests; categorical variables are analyzed with Pearson chi-square tests or Mann-Whitney U tests, as appropriate. BMI: body mass index; ASA physical status: American Society of Anesthesiologists physical status; CEA: carcinoembryonic antigen; pRBC: packed red blood cell; C/T: chemotherapy; TT: target therapy; R/T: radiotherapy.

**Table 2:** Cancer staging and pathologic features.

	EA (n = 165)	Non-EA (n = 834)	*p*
**AJCC stage**			0.406
Stage IVa	97 (58.8%)	461 (55.3%)	
Stage IVb	68 (41.2%)	373 (44.7%)	
**Isolated liver metastases**	70 (42.4%)	300 (36.0%)	0.117
**Pathologic features**			
Tumor differentiation			0.557
Well- or Moderately-differentiated	140 (88.6%)	683 (86.9%)	
Poorly- or Un-differentiated	18 (11.4%)	103 (13.1%)	
Mucinous histology	8 (5.1%)	65 (8.3%)	0.166
Signet-ring histology	5 (3.2%)	37 (4.7%)	0.388
Lymphovascular invasion	70 (44.3%)	415 (52.7%)	0.053
Perineural invasion	24 (15.2%)	184 (23.5%)	0.022

Values were counts (percent). Categorical variables are analyzed with Pearson chi-square tests or Mann-Whitney U tests, as appropriate. AJCC: American Joint Committee on Cancer

### Association between EA and cumulative progression rate

1-yr and 2-yr cumulative incidence of progression were 64.2% (95% CI: 56.4–72.0%) and 87.2% (95% CI: 81.5–92.9%) in the EA group and 68.1% (95% CI: 64.8–71.4%) and 86.6% (95% CI: 84.1–89.1%) in the non-EA group. Median progression-free survival time was 8.4 months (95% CI: 6.7–10.1 months) in the EA group and 7.4 months (95% CI: 6.6–8.1 months) in the non-EA group. For patients with isolated hepatic metastases, 1-yr and 2-yr cumulative incidence of progression were 21.6% and 40.9% in the EA group and 23.2% and 44.4% in the non-EA group. No significant difference in the median progression-free survival time was noted between the two groups (28.2 months in the EA group vs. 28.6 months in the non-EA group, *p* = 0.62).

**Fig 2:**
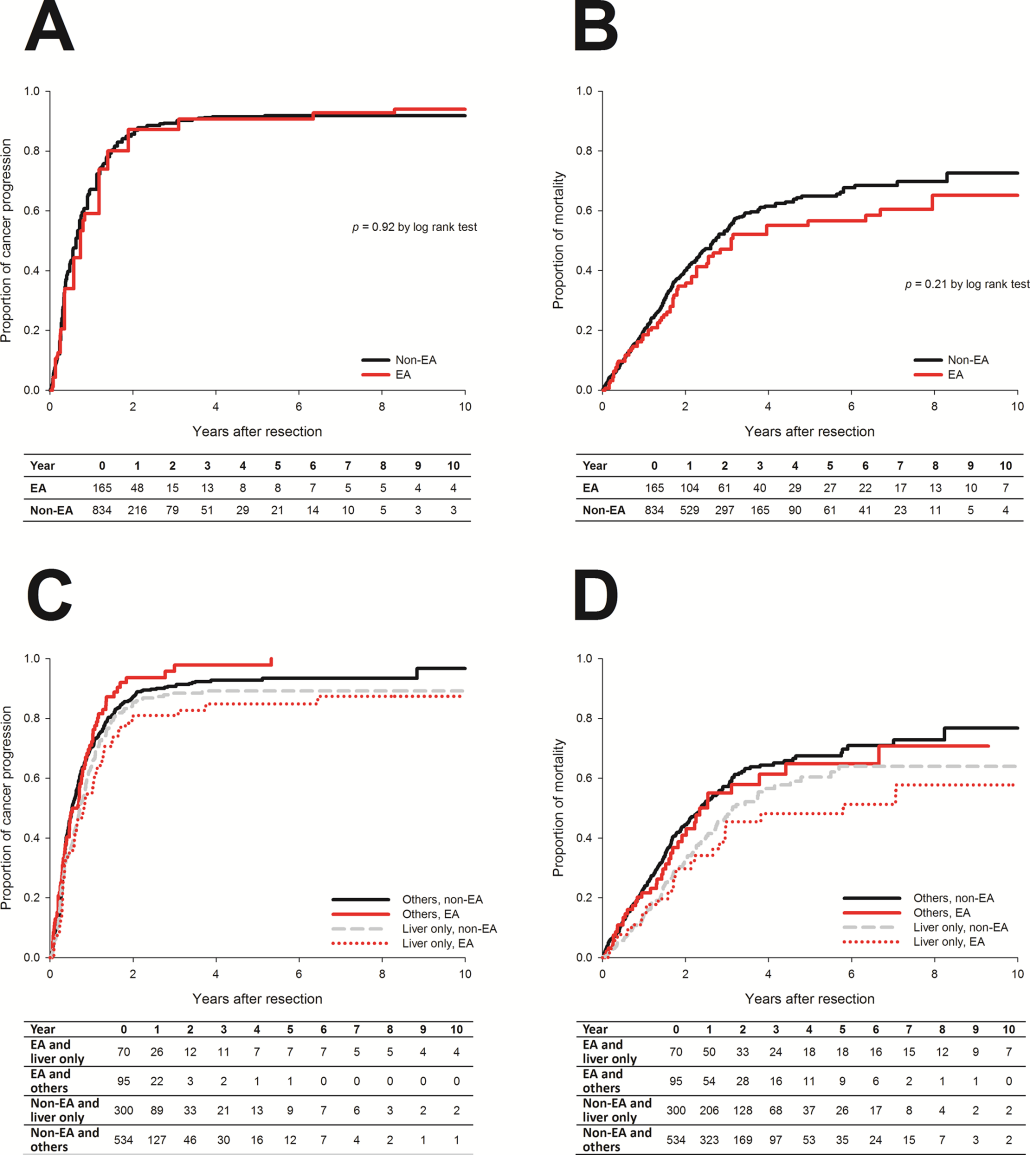
Cumulative incidences of cancer progression and all-cause mortality between EA and non-EA groups. No significant difference in cancer progression (Fig 2A and 2B) or overall mortality (Figs 2C and 2D) after surgery for stage IV colorectal cancer was noted when comparing EA with non-EA groups.

No significant difference in the distribution of progression-free survival was found when comparing EA with non-EA groups (HR: 0.990, 95% CI: 0.821–1.194, *p* = 0.92 by log rank test, Fig 2A). Subgroup analyses also revealed that patients with or without isolated hepatic metastases showed no significant difference in disease progression between EA and non-EA groups (log rank test: *p* = 0.62 for isolated hepatic metastases; *p* = 0.24 for extrahepatic metastases, Fig 2C). Univariate analysis identified several significant risk factors of cancer progression, including higher pretreatment CEA, multiple distant metastases (i.e. stage IVb disease), extrahepatic metastases, shorter anesthesia time, specific pathologic findings (lymphovascular invasion and perineural invasion), preoperative chemotherapy and/or radiotherapy, and postoperative chemotherapy. (Table 3) Note that EA was not associated with cancer progression after surgery in the univariate analysis (HR: 0.99, 95% CI: 0.82–1.19).

**Table 3:** Univariate analysis of cancer progression and all-cause mortality.

	Cancer progression			All-cause mortality		
	HR	95% C.I.	*p*	HR	95% C.I.	*p*
**EA vs. non-EA**	0.990	0.821–1.194	0.918	0.847	0.652–1.101	0.214
**Age**	0.998	0.992–1.003	0.399	1.009	1.002–1.017	0.016
**Sex (F vs. M)**	1.001	0.866–1.156	0.993	1.008	0.831–1.224	0.935
**BMI**	0.979	0.958–1.001	0.067	0.954	0.925–0.984	0.003
**ASA ≥ 3**	1.147	0.992–1.328	0.065	1.563	1.289–1.895	< 0.001
**Diabetes**	0.852	0.714–1.018	0.078	1.033	0.820–1.301	0.784
**Coronary arterial disease**	0.878	0.666–1.158	0.359	0.801	0.549–1.168	0.248
**Heart failure**	1.153	0.809–1.643	0.430	1.514	0.994–2.305	0.054
**Stroke**	1.114	0.807–1.538	0.510	1.199	0.812–1.772	0.361
**Chronic kidney disease**	1.116	0.906–1.375	0.300	1.334	1.029–1.729	0.029
Pretreatment CEA*	1.295	1.195–1.404	< 0.001	1.572	1.416–1.746	< 0.001
**pRBC transfusion**			0.735			< 0.001
≦ 4 units vs. nil	1.019	0.870–1.193	0.818	1.267	1.024–1.568	0.029
> 4 units vs. nil	1.097	0.870–1.383	0.433	2.160	1.648–2.833	< 0.001
Anesthesia time**	0.834	0.721–0.965	0.015	0.796	0.659–0.961	0.018
**Preoperative C/T ± R/T**	1.276	1.056–1.542	0.012	1.018	0.777–1.333	0.898
**Postoperative C/T**			< 0.001			< 0.001
Pure C/T vs. nil	1.242	0.863–1.788	0.244	0.282	0.202–0.394	< 0.001
C/T + TT vs. nil	1.821	1.270–2.609	0.001	0.316	0.229–0.435	< 0.001
**Postoperative R/T**	1.215	0.986–1.497	0.068	1.022	0.774–1.348	0.880
**Tumor location**			0.197			0.038
Left vs. right-sided	1.044	0.879–1.240	0.626	0.898	0.720–1.120	0.341
Rectum vs. right-sided	1.173	0.977–1.409	0.086	0.721	0.560–0.928	0.011
**Stage IVb vs. IVa**	1.756	1.521–2.027	< 0.001	2.502	2.058–3.041	< 0.001
**Isolated liver metastases**	0.801	0.692–0.928	0.003	0.692	0.565–0.846	< 0.001
**Tumor differentiation**^**a**^	1.210	0.972–1.505	0.088	1.798	1.354–2.388	< 0.001
**Mucinous histology**	0.939	0.715–1.233	0.651	0.946	0.635–1.409	0.785
**Signet-ring histology**	0.955	0.663–1.377	0.807	1.393	0.878–2.209	0.159
**Lymphovascular invasion**	1.450	1.254–1.677	< 0.001	1.720	1.407–2.102	< 0.001
**Perineural invasion**	1.520	1.281–1.805	< 0.001	1.530	1.213–1.928	< 0.001

HR: hazard ratio; EA: epidural analgesia; F: female, M: male; BMI: body mass index; ASA physical status: American Society of Anesthesiologists physical status; CEA: carcinoembryonic antigen; pRBC: packed red blood cell; C/T: chemotherapy; TT: target therapy; R/T: radiotherapy.

* On base-10 logarithmic scale

** On base-2 logarithmic scale

^a^ Poorly- or Un-differentiated vs. Well- or Moderately-differentiated tumors

After the model selection processes, eight independent risk factors were identified after multivariable analysis, including ASA ≥ 3, higher pretreatment CEA, shorter anesthesia time, multiple distant metastases, pathologic lymphovascular invasion, preoperative chemotherapy and/or radiotherapy, postoperative chemotherapy and postoperative radiotherapy. (Table 4) After the adjustment for these independent predictors, the effect of EA on cancer progression after surgery for stage IV CRC remains non-significant (HR: 1.06, 95% CI: 0.87–1.29, *p* = 0.55). Furthermore, both the covariate-adjusted (HR: 1.01, 95% CI: 0.83–1.22, *p* = 0.96) and quintile-stratified propensity score analyses (Pooled HR: 0.99, 95% CI: 0.81–1.21, *p* = 0.92) demonstrated no significant association between EA and stage IV CRC progression after surgery.

**Table 4:** Forward model selection for progression-free and overall survival.

	HR	95% C.I.	*p*
**Progression-free survival**			
ASA ≥ 3	1.232	1.055–1.439	0.008
Pretreatment CEA *	1.212	1.116–1.316	< 0.001
Anesthesia time **	0.828	0.708–0.970	0.019
Preoperative C/T ± R/T	1.381	1.131–1.687	0.002
Postoperative C/T			< 0.001
Pure C/T vs. nil	1.251	0.861–1.817	0.241
C/T + TT vs. nil	1.673	1.154–2.425	0.007
Postoperative R/T	1.260	1.011–1.570	0.039
Stage IVb vs. IVa	1.641	1.408–1.912	< 0.001
Lymphovascular invasion	1.317	1.134–1.531	< 0.001
EA vs. non-EA	1.063	0.873–1.294	0.545
**Overall survival**			
ASA ≥ 3	1.417	1.148–1.749	0.001
Pretreatment CEA *	1.550	1.388–1.731	< 0.001
pRBC transfusion			0.002
≦ 4 units vs. nil	1.140	0.913–1.424	0.248
> 4 units vs. nil	1.727	1.276–2.337	< 0.001
Postoperative C/T			< 0.001
Pure C/T vs. nil	0.384	0.262–0.564	< 0.001
C/T + TT vs. nil	0.343	0.235–0.500	< 0.001
Stage IVb vs. IVa	2.192	1.770–2.715	< 0.001
Tumor differentiation^a^	1.641	1.216–2.215	0.001
Lymphovascular invasion	1.489	1.204–1.841	< 0.001
EA vs. non-EA	0.904	0.683–1.197	0.483

HR: hazard ratio; CEA: carcinoembryonic antigen; C/T: chemotherapy; TT: target therapy; R/T: radiotherapy; EA: epidural analgesia; ASA physical status: American Society of Anesthesiologists physical status; pRBC: packed red blood cell.

* On base-10 logarithmic scale

** On base-2 logarithmic scale

^a^ Poorly- or Un-differentiated vs. Well- or Moderately-differentiated tumors

### Association between epidural analgesia and cumulative mortality rate

1-yr and 2-yr overall mortality rate were 18.5% (95% CI: 12.2–24.8%) and 35.8% (95% CI: 27.2–44.4%) in the EA group and 20.0% (95% CI: 17.1–22.9%) and 40.2% (95% CI: 36.3–44.1%) in the non-EA group. Median survival time in the EA group was 35.7 months (95% CI: 21.3–50.0 months); in the non-EA group, median survival time was 32.0 months (95% CI: 28.5–35.6 months). For patients with isolated hepatic metastases, 1-yr and 2-yr overall mortality rate were 14.3% and 29.8% in the EA group and 14.4% and 33.0% in the non-EA group. No significant difference in median survival time was noted between the two groups (69.6 months in the EA group vs. 37.7 months in the non-EA group, *p* = 0.37. No significant reduction in overall mortality after surgery was found when comparing EA with non-EA groups (*p* = 0.21 by log rank test, Fig 2B). Stratified analysis showed that there was no significant difference in overall mortality between groups, no matter whether patients had isolated liver metastases or not (log rank test: *p* = 0.37 for isolated hepatic metastases; *p* = 0.31 for extrahepatic metastases, Fig 2D). Univariate analysis disclosed several significant risk factors of overall mortality, including older age, lower BMI, ASA physical status ≥ 3, higher pretreatment CEA, chronic kidney disease, perioperative pRBC transfusion, shorter anesthesia time, right-sided colon cancer, multiple distant metastases, extrahepatic metastases, specific pathologic findings (poorly- or un-differentiation, lymphovascular invasion, and perineural invasion), and absence of postoperative chemotherapy. (Table 3)

Seven independent prognostic determinants were identified after multivariable analysis, including ASA physical status ≥ 3, higher pretreatment CEA, multiple distant metastases, perioperative pRBC transfusion > 4 units, poorly- or un-differentiated tumor, pathologic lymphovascular invasion and postoperative chemotherapy. (Table 4) Note that after the adjustment for these predictors, the effect of EA on overall mortality after surgery for stage IV CRC remains non-significant (HR: 0.90, 95% CI: 0.68–1.20, *p* = 0.48). In addition, both the covariate-adjusted (HR: 0.89, 95% CI: 0.67–1.18, *p* = 0.42) and quintile-stratified propensity score analytical methods obtained compatible results with the multivariable regression analysis (HR: 0.89, 95% CI: 0.67–1.18, *p* = 0.43).

## Discussion

This retrospective study did not confirm a definite association between EA and cancer progression or overall survival in patients following primary tumor and metastatic lesion resection for stage IV colorectal cancer. It has been proposed that opioids promote tumor growth and metastasis through the pathway of activated mu-opioid receptor (MOR) [8, 22]. Preclinical studies showed mu opioids may inhibit interleukin and natural killer cell activity and enhance tumorigenicity [23, 24]. Zylla and colleagues reported that greater opioid requirements were associated with shorter PFS and OS in patients with advanced prostate cancer [25]. MORs have also been demonstrated in the nuclei of human colon cancer cells, and exposure of these cells to morphine increased secretion of urokinase plasminogen activator, a promoter of tumor invasion and metastasis [26]. Although some laboratory findings supported the potential benefit of EA to cancer outcomes after surgery, there is still a gap between laboratory results and clinical evidence. To date, only two reports examined the associations between EA and oncologic outcomes in patients with metastatic CRC. EA was reported to be associated with better survival in patients with non-metastatic CRC, but no effect on survival of patients with metastases was observed [27]. However, the report was limited by small sample size (only 65 patients of stage III or IV disease) and mixed groups of patients for nodal and distant metastases. A recent study suggested an association between EA and improved recurrence-free survival (multivariable analysis HR: 0.74, 95% CI: 0.56–0.95, *p* = 0.036), but not overall survival, after colorectal liver metastases resection [21], which stands in contrast to our results. This may be explained by the difference in disease severity (only 5.5% with multiple distant metastases versus 44.1% in our sample) and adjuvant treatment (83.3% with preoperative chemotherapy versus 15.5% in our subjects). The major strength of our study was taking critical pathologic and prognostic factors into accounts and adjusting their effects to eliminate potential impact of these confounders from the evaluation of the relationship between EA and PFS or OS in patients following surgery for stage IV CRC.

Among patients with stage IV CRC, although prognosis may be closely tied to the location and extent of distant metastatic disease, our findings suggested other important clinicopathologic predictors. Lymphovascular invasion has been proved to be a pathologic predictor for poor outcomes in CRC [14, 28] and included in the definition of high-risk stage II CRC from the American Society of Clinical Oncology (ASCO) [29] and European Society for Medical Oncology (ESMO) [30]. Our study demonstrated lymphovascular invasion is also a significant risk factor for disease progression and overall mortality in stage IV CRC. Besides, undergoing preoperative chemotherapy and/or radiotherapy were associated with shorter PFS, which may merely reflect a more advanced disease at the time of surgery. Preoperative serum levels of the tumor marker CEA are of prognostic significance. CEA levels ≥ 5.0 ng·mL^-1^ have an adverse impact on survival that is independent of tumor stage [12, 31]. Within each stage grouping, the prognosis of the subset of patients with elevated CEA was similar to or worse than a subset of patients with a higher AJCC stage grouping with a normal pretreatment CEA level [12]. Higher CEA level at baseline also independently predicts worse survival in metastatic CRC [32]. Furthermore, our analysis also revealed that pretreatment CEA level was also a significant prognostic factor of disease progression in stage IV CRC.

Our results implicated perioperative pRBC transfusion was linked to shorter overall survival and there existed a dose-response relationship. Meta-analyses demonstrated that perioperative blood transfusions have a detrimental effect on the cancer recurrence and long-term mortality in patients undergoing surgery of curable colorectal cancers [33]. Recent studies reported that perioperative transfusion is independently associated with earlier disease recurrence and decreased overall survival in patients undergoing liver resection for colorectal liver metastases [34]. Administration of blood products exerts negative impacts on the human immune system, including suppression of cytotoxic cell activity, release of immunosuppressive prostaglandins, inhibition of interleukin-2 production, and increase in suppressor T-cell activity [35]. However, a recent trial reported that transfusion reduction initiative did not prolong colorectal cancer disease-free survival [36]. The association between transfusion and cancer outcomes after surgery deserved more investigations.

Prior studies reported right-sided tumors have worse cancer prognosis than left-sided tumors in metastatic CRCs [37]. Cancer genomic studies indicated that proximal (right-sided) and distal CRCs (left-sided) follow different molecular pathways of carcinogenesis. Microarray studies of sporadic CRC biopsies demonstrate differences in gene expression between adenocarcinomas of the cecum and sigmoid or rectosigmoid [38]. Right-sided tumors are more likely to be diploid and characterized by high microsatellite instability and BRAF mutations [39, 40]. Although our univariate analysis suggested right-sided tumor was associated with shorter overall survival, no significant difference in mortality was seen after adjusting for other significant risk factors. Perhaps, the relationship between tumor location and colorectal cancer outcomes is not straightforward. More research was needed to elucidate the issue.

Several important limitations are inherent in this retrospective and observational study. Patients were not randomized and clinical care was not standardized, so that selection bias and the effects of unmeasured confounding variables cannot be excluded. Second, the data of total narcotic requirements, perioperative analgesics, and intraoperative chemotherapy (e.g. hyperthermic intraperitoneal chemotherapy) for each patient could not be obtained due to the limitation of data requisition. Third, a sizable portion of patients did not receive EA due to technical difficulty or contraindications. Although this may introduce selection bias, we found there were only minor differences in the distributions of patient attributes and pathologic outcomes which were further adjusted in the multivariable analyses and these should not be serious issues to interfere with the accuracy of estimation. Fourth, the disease in stage IV CRC might be too advanced for an epidural to exert its protective effect on cancer outcomes.

However, compared with previous studies investigating similar issues [21, 27], our study has the strength of larger sample size and more comprehensive collection of clinicopathologic predictors, which provided new and more solid evidence to challenge the controversial relationship between EA and postoperative outcomes of stage IV colorectal cancer.

Our results did not support the association between perioperative epidural analgesia and better progression-free or overall survival in patients with stage IV colorectal cancer following primary tumor and metastatic lesion resection. The clinical benefit of EA to patients receiving surgical resection for metastatic colorectal cancer awaits further investigation.

## Supporting information

S1 Stage 4 Dataset. Dataset for the analysis of progression-free and overall survival time of stage IV colorectal cancer.
(XLS)
