# Correction: The effect of epidural analgesia on cancer progression in patients with stage IV colorectal cancer after primary tumor resection: A retrospective cohort study

**DOI:** 10.1371/journal.pone.0202192

**Published:** 2018-08-07

**Authors:** Ying-Hsuan Tai, Wen-Kuei Chang, Hsiang-Ling Wu, Min-Ya Chan, Hsiu-Hsi Chen, Kuang-Yi Chang

The third affiliation for the second author is not indicated. Wen-Kuei Chang is also affiliated with: Taipei Municipal Gan-Dau Hospital, Taipei, Taiwan.
